# Treatment Outcomes of 9,994 Patients With Extensive-Disease Small-Cell Lung Cancer From a Retrospective Nationwide Population-Based Cohort in the Korean HIRA Database

**DOI:** 10.3389/fonc.2021.546672

**Published:** 2021-03-22

**Authors:** Jung Soo Lee, Seoree Kim, Soo-Yoon Sung, Yeo Hyung Kim, Hyun Woo Lee, Ji Hyung Hong, Yoon Ho Ko

**Affiliations:** ^1^ Department of Rehabilitation Medicine, College of Medicine, The Catholic University of Korea, Seoul, South Korea; ^2^ Division of Oncology, Department of Internal Medicine, College of Medicine, The Catholic University of Korea, Seoul, South Korea; ^3^ Department of Radiation Oncology, College of Medicine, The Catholic University of Korea, Seoul, South Korea; ^4^ Department of Hematology-Oncology, Ajou University School of Medicine, Suwon, South Korea; ^5^ Cancer Research Institute, College of Medicine, The Catholic University of Korea, Seoul, South Korea

**Keywords:** efficacy, systemic chemotherapy, population-based cohort study, prognosis, extensive-disease small cell lung cancer

## Abstract

To investigate the efficacy of irinotecan-based (IP) and etoposide-based (EP) platinum combinations, and of single-agent chemotherapy, for treatment of extensive-disease small cell lung cancer (ED-SCLC), we performed a large-scale, retrospective, nationwide, cohort study. The population data were extracted from the Health Insurance Review and Assessment Service of Korea database from January 1, 2008, to November 30, 2016. A total of 9,994 patients were allocated to ED-SCLC and analyzed in this study. The primary objectives were to evaluate the survival outcomes of systemic first-line treatments for ED-SCLC. For first-line treatment, patients who received IP showed a better time to first subsequent therapy (TFST) of 8.9 months (95% confidence interval [CI], 8.50–9.40) than those who received EP, who had a TFST of 6.8 months (95% CI, 6.77–6.97, P < 0.0001). In terms of overall survival (OS), IP was superior to EP (median OS, 10.8 months; 95% CI, 10.13–11.33 vs. 9.5 months; 95% CI, 9.33–9.73; P < 0.0001). Taken together, in the Korean population, first-line IP combination chemotherapy had significantly favorable effects on OS and TFST.

## Introduction

Lung cancer is the main cause of cancer-related death worldwide, and the small cell lung cancer (SCLC) subtype includes only 11%–14% of total lung cancer diagnoses (1–3). Biologically, SCLC is aggressive lung cancer subtype, with a high frequency of metastasis and early dissemination. At diagnosis, more than two-thirds of patients have extensive-disease (ED) SCLC. The majority of patients with ED-SCLC die within 1 year of initial diagnosis due to relapse, despite the initial sensitivity of platinum-based chemotherapy (2).

Platinum-based chemotherapy including etoposide or irinotecan can produce a 60–80% response rate (RR) and 7–12 months of median survival in patients with ED-SCLC (4). However, despite good response, improvement during the past decade has been limited; the 2-year survival rate increased only from 3.4% to 5.6% (5). Etoposide with platinum (EP) is currently the standard first-line treatment used to obtain longer overall survival (OS) and progression-free survival (PFS) in Western populations; however, it results in a only 2% 5-year survival rate (6). In contrast, subsequent Eastern Asian studies have yielded contradictory results. A Japanese phase III study, comparing the efficacy of irinotecan with cisplatin (IP) versus EP as first-line chemotherapy, showed improved survival for IP compared to EP (7). However, the following trials did not support the superiority of IP over EP (8, 9). In a recent phase III study, first-line IP also did not significantly improve survival compared to EP (2). Also, there is no established consensus regarding the most effective second-line regimen. Especially in Korea, there is a tendency to use a less toxic single agent rather than a platinum-based combination because of patients’ poor performance and organ dysfunction (10–12). Therefore, determining the clinical efficacy of first- and second-line systemic therapies in a larger population would enable treatment strategies for ED-SCLC to be refined.

To date, no large-scale studies have assessed the efficacy of each systemic regimen in ED-SCLC patients in an East Asian population. Korean health insurance covers the entire population of Korea, and the Health Insurance Review and Assessment Service of Korea (HIRA) provides information on healthcare services provided to the Korean population. Thus, using the HIRA database, we could approach the entire Korean population and analyze the efficacy of systemic chemotherapy in a large population of patients with ED-SCLC who received palliative systemic treatment.

## Materials and Methods

### Study Design

**Figure 1:**
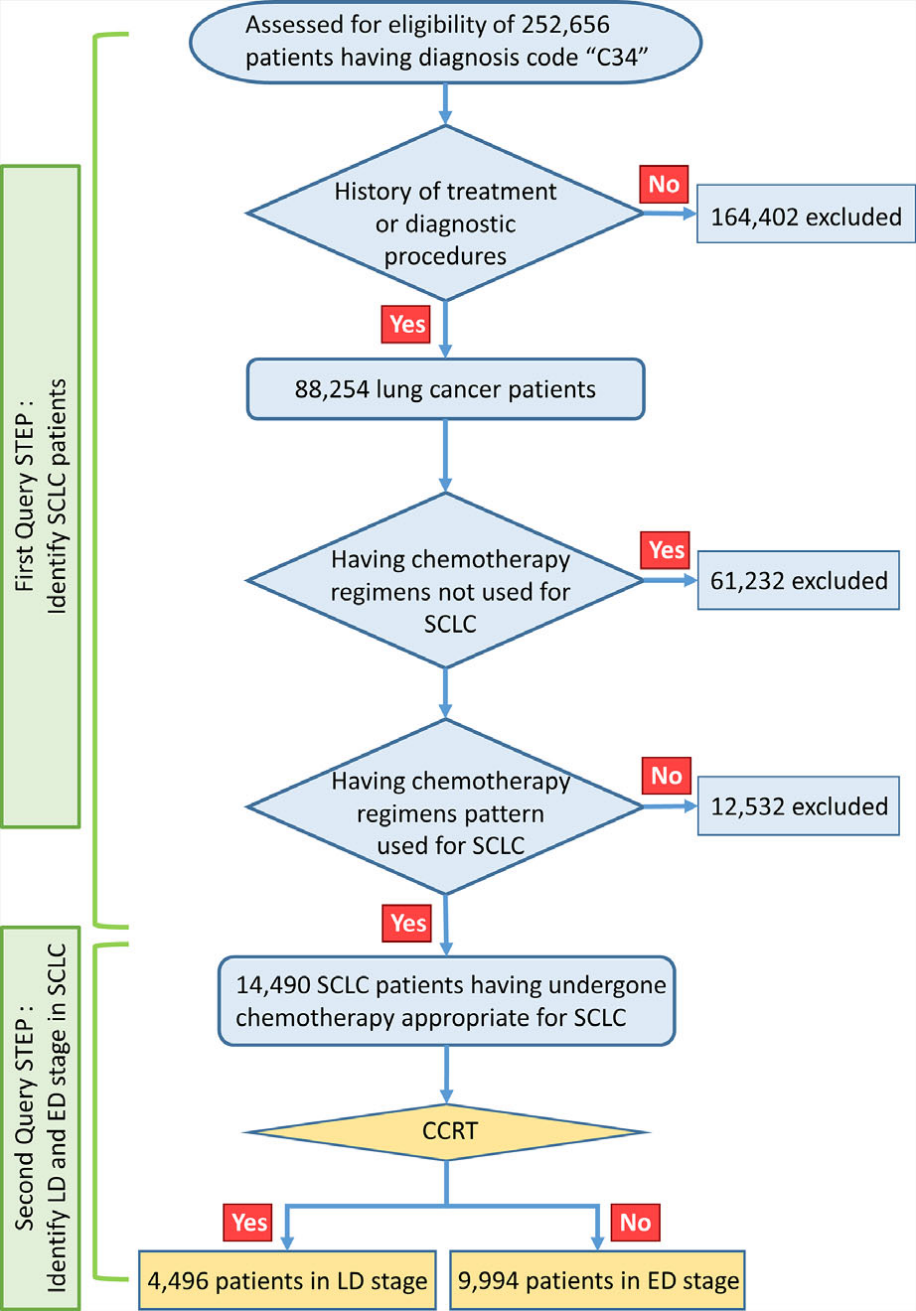
Study design.

This large-scale, retrospective, nationwide cohort study was approved by the institutional review board of the Uijeongbu St. Mary Hospital and HIRA (No. UC18ZESI0145). The requirement for written informed consent was waived because this study retrospectively analyzed national insurance cohort data. The data-mining scheme used in this study is shown in **Figure 1**. The HIRA data include all ICD-10 diagnostic codes and billing codes for all medical services, such as diagnostic procedures and treatment modalities (such as drug prescriptions, radiotherapy, or surgery), provided to the entire population of Korea. We performed data mining using a query program to classify the appropriate SCLC patient cohort.

### Study Population

A total of 252,656 patients were identified as having C34 ICD-10 diagnostic code in the HIRA database from January 1, 2008, to March 31, 2018. 238,166 were excluded, as they had received chemotherapy regimens only used for NSCLC such as pemetrexed, gemcitabine, docetaxel, vinorelbine, epidermal growth factor receptor (EGFR) tyrosine kinase inhibitor (TKI), or anaplastic lymphoma kinase (ALK) TKI, or had other types of cancer. In the first query step, billing codes were used to identify SCLC; these consisted of chest radiotherapy and drugs such as belotecan, irinotecan, etoposide, vincristine, ifosfamide, cyclophosphamide, topotecan, cisplatin, carboplatin, doxorubicin, and paclitaxel, which are covered for ED-SCLC by the Korean national health insurance service. SCLC patients were defined and classified according to the types and orders of use of chemotherapeutic regimens for each stage of SCLC defined by the national health insurance service regulations of Korea. The remaining 14,490 patients were selected as having undergone chemotherapy appropriate for SCLC. The operational definition of patients with limited disease (LD) SCLC was those who received definitive concurrent chemoradiotherapy (CCRT); otherwise, the patient was considered to have ED-SCLC. A total of 4,496 patients with LD-SCLC were defined using the operational criteria, while the remaining 9,994 patients were allocated to ED-SCLC and analyzed in this study. To verify the reliability of the operational criteria for SCLC staging, we used single-institution data from 357 SCLC patients with known disease status. Using these operational criteria, patients with ED-SCLC were predicted with a sensitivity of 100%, specificity of 64.6%, and accuracy of 88.5% (**Supplementary Table 1**).

In patients with LD-SCLC defined by our operational criteria, the median survival duration was 21.8 months (95% confidence interval [CI], 20.86–22.96); in patients with ED-SCLC, 9.6 months (95% CI, 9.43–9.83, **Supplementary Figure 1**). Five-year survival rates were 24.73 ± 0.75% and 8.13 ± 0.30%, respectively. These findings are comparable with the survival outcomes of LD and ED-SCLC patients in recent studies (13–15). Thus, our operational criteria were considered acceptable.

### Definition of Survival Outcomes

The time to first subsequent therapy (TFST) duration was defined as the time from the date of first-line chemotherapy until subsequent chemotherapy or death due to any cause, whichever was observed first. The overall survival (OS) duration was calculated from the date of diagnosis to the date of death or the last follow-up visit. The date of diagnosis was defined as the date when first chemotherapy or surgery or radiotherapy was started after the first application of the C34 diagnostic code.

### Statistical Analysis

The primary objectives were to evaluate the survival outcomes of systemic first-line treatments for ED-SCLC. The secondary objectives were to evaluate the survival outcomes of the regimens as second-line treatments. Baseline characteristics are presented as means (± standard error) and medians (ranges) for continuous variables and frequencies (%) for categorical variables. A *t*-test was performed for comparisons of continuous variables, and Pearson’s chi-squared test or a two-sample proportion z-test for comparisons of categorical variables. We performed a Cox proportional hazards regression to identify the risk factors for overall mortality, because the Cox proportional hazards assumption was satisfied for the variables analyzed in this study. The survival curves were estimated using the Kaplan–Meier method and compared using the log-rank test. SAS Enterprise Guide version 6.1 (SAS Inc., Cary, NC, USA), Visual Basic for Applications 7.0 (Microsoft Inc., Redmond, WA, USA), and Excel 2010 (Microsoft Inc., Redmond, WA, USA) were used to perform all data mining and statistical analyses.

## Results

### Baseline Characteristics

A total of 9,994 patients were analyzed as having ED-SCLC. Their demographic features are shown in **Table 1**. The mean age was 68 years. As first-line treatment, 9,618 patients received combination chemotherapy (combination chemotherapy group [CG]), and the remaining 376 received a single agent (single agent group [SG]). The most common first-line regimen was an etoposide with platinum combination. For the second-line regimen, combination chemotherapy was used in 2,213 patients and single-agent chemotherapy in 2,085. Irinotecan combined with platinum in CG and belotecan in SG were the most frequently used second-line treatment regimens.

**Table 1:** Demographic characteristics of 9,994 patients with ED-SCLC with systemic chemotherapy.

	Total (n = 9,994)
Age	68 (SD 8.4)
Gender (Male/Female)	8,634 (86.4%)/1,360 (13.6%)
Comorbidities	
HBP	5,677 (56.8%)
DM	2,719 (27.2%)
Dyslipidemia	4,623 (46.3%)
COPD	2,015 (20.2%)
First-line chemotherapy	
Combination chemotherapy	9,618 (96.2%)
Etoposide/platinum	8,142 (81.4%)
Irinotecan/platinum	1,476 (14.8%)
Single-agent chemotherapy	376 (3.8%)
Etoposide	213 (2.1%)
Irinotecan	71 0.7%)
Belotecan	92 (0.9%)
Second-line chemotherapy	
Combination chemotherapy	2,123 (21.2%)
Etoposide/platinum	598 (6.0%)
Irinotecan/platinum	1,525 (15.3%)
Single-agent chemotherapy	2,085 (20.8%)
Etoposide	31 (0.3%)
Irinotecan	561 (5.6%)
Belotecan	920 (9.2%)
Topotecan	573 (5.7%)

ED-SCLC, extensive-disease small-cell lung cancer; SD, standard deviation; HBP, hypertension; DM, diabetes mellitus; COPD, chronic obstructive pulmonary disease.

### Survival Outcomes With the First-Line Treatment

**Figure 2:**
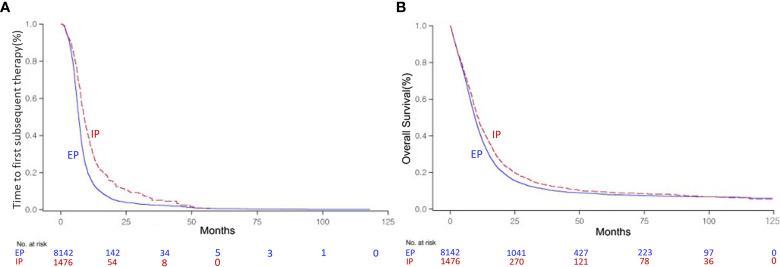
Kaplan–Meier curve of the time to first subsequent therapy (TFST) **(A)** and overall survival (OS) **(B)** of the irinotecan/platinum (IP) and etoposide/platinum (EP) combinations as first-line regimens for extensive-disease (ED) small-cell lung cancer (SCLC).

Within a 9.6-month median follow-up period, analysis of the survival data revealed 8,907 death (89.1%) events in the ED group of 9,994. In CG, of note, the IP combination showed significantly better TFST of 8.9 months (95% CI, 8.50–9.40) than the EP combination at 6.8 months (95% CI, 6.77–6.97) (P < 0.0001, **Figure 2A**). In terms of OS, significantly improved survival benefit was also found in patients with the IP combination at 10.8 months (95% CI, 10.13–11.33) compared with the EP combination at 9.5 months (95% CI, 9.33–9.73) (P < 0.0001, **Figure 2B**). The median TFST time was 7.1 months (95% CI, 6.70–7.23) in the CG group and 6.1 months (95% CI, 5.37–6.77) in the SG group (P < 0.0001, **Supplementary Figure 2A**). The median OS time was 9.7 months (95% CI, 9.50–9.90) in the CG group and 7.3 months (95% CI, 6.23–8.53, P < 0.0001) in the SG group (**Supplementary Figure 2B**). In SG, there were no significant differences among the three monotherapies for TFST (P = 0.4101). Belotecan showed better OS than etoposide or irinotecan monotherapy (14.7 months, 95% CI, 12.83–17.00 vs. 4.16 months, 95% CI, 3.06–5.56 vs. 6.66 months, 95% CI, 5.26–8.53, respectively, P < 0.0001, **Supplementary Figure 2C**).

### Survival Outcomes With the Second-Line Treatment

**Figure 3:**
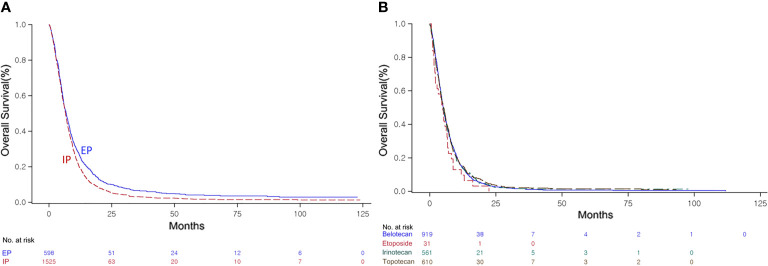
Kaplan–Meier curve of the overall survival (OS) of the irinotecan/platinum (IP) and etoposide/platinum (EP) combinations **(A)**, and the OS of the single agents **(B)** as second-line regimens.

Following failure of first-line chemotherapy, the combination chemotherapy in the second line demonstrated significantly improved OS of 6.6 months (95% CI, 6.36–6.96) compared with the single regimens at 5.1 months (95% CI, 4.93–5.36) (P < 0.0001, **Supplementary Figure 3A**). In patients with SCLC who failed or relapsed after first-line EP chemotherapy, a similar finding was also observed (SG: 5.1 months, 95% CI, 4.93–5.36 vs. CG: 6.5 months, 95% CI, 6.23–6.86, P < 0.0001, **Supplementary Figure 3B**). Unlike with first-line treatment, the EP combination showed significantly better OS of 6.9 months (95% CI, 6.13–7.53) than the IP combination at 6.6 months (95% CI, 6.30–6.90) (P = 0.0009, **Figure 3A**). However, OS did not differ significantly among single-agent regimens as second-line treatment (P = 0.5856, **Figure 3B**).

### Factors Associated With Survival Outcomes in Patients With Extensive-Disease Small Cell Lung Cancer

The univariate analyses demonstrated that elder age, male gender, hypertension, chronic obstructive pulmonary disease (COPD), absence of hypercholesterolemia and diabetes mellitus, EP combination compared with the IP combination were significantly associated with shorter OS (**Table 2**). In the multivariate Cox proportional hazards regression analysis, all factors retained their independence toward OS. Also, the EP combination was significantly associated with poorer OS (adjusted odds ratio [OR], 1.18, 95% CI, 1.10–1.27; P < 0.0001) compared to IP as first-line treatment.

**Table 2:** Relative risk for overall survival of 9,994 patients with ED-SCLC.

	Unadjusted OR (95% CI)	P-value	Adjusted OR (95% CI)	P-value
Age	1.52 (1.42–1.62)	<0.0001	1.47 (1.37–1.57)	<0.0001
Gender (**male** vs. female)	1.19 (1.09–1.30)	<0.0001	1.19 (1.09–1.30)	<0.0001
HBP (**HBP** vs. normal)	1.08 (1.01–1.14)	0.0103	1.07 (1.00–1.14)	0.03
DM (**normal** vs. DM)	1.13 (1.06–1.21)	0.0002	1.12 (1.04–1.20)	0.001
Hypercholesterolemia (**normal** vs. hypercholesterolemia)	1.08 (1.02–1.15)	0.005	1.11 (1.04–1.18)	0.001
COPD (**COPD** vs. normal)	1.25 (1.16–1.34)	<0.0001	1.17 (1.09–1.26)	<0.0001
1st line chemotherapy regimen (reference, irinotecan/platinum combination)				
Belotecan	1.05 (0.81–1.35)	0.7114	1.04 (0.80–1.34)	0.75
Etoposide	2.37 (1.98–2.84)	<0.0001	2.25 (1.88–2.69)	<0.0001
Irinotecan	1.34 (1.02–1.75)	0.0337	1.38 (1.05–1.81)	0.02
Etoposide/platinum	1.18 (1.09–1.26)	<0.0001	1.18 (1.10–1.27)	<0.0001

ED-SCLC, extensive-disease small-cell lung cancer; OR, odds ratio; 95% CI, 95% confidence interval; HBP, hypertension; DM, diabetes mellitus; COPD, chronic obstructive pulmonary disease. Variables with odds ratio are shown in bold type.

## Discussion

In this study, the survival outcomes of patients with ED-SCLC were better among those who received the IP regimen than those who received the EP regimen in the first-line setting. If a single agent was required, despite inferior tumor control to platinum-based combination chemotherapy, the OS of patients who received belotecan as the first-line treatment was better than that of those administered irinotecan or etoposide alone. In the second-line setting, EP had a better OS than IP, unlike the first-line setting. The single-agent chemotherapies as the second-line treatments did not significantly differ in terms of OS. This study provides evidence that the irinotecan and platinum combination as the first-line therapy may be the gold standard first-line regimen for Korean patients with ED-SCLC. To the best of our knowledge, this analysis includes the largest study population to date.

Nowadays, based on IMpower 133 and Caspian trial, atezolizumab or durvalumab combined platinum-based doublet chemotherapy have been updated as the standard of care in the first-line regimen of extensive disease of SCLC (16, 17). However, over the past 20 years, standard therapy for most patients with ED-SCLC has been a platinum-based etoposide combination regimen. In 2002, in the Japanese Clinical Oncology Group (JCOG)-9511 phase III study, which compared EP to IP, the tumor response and patient survival outcomes were significantly better in the IP group at the interim analysis, prompting early termination of further accrual (7). Because of the small number of patients (n = 174), the study involved a solely Japanese population. Subsequently, a phase III trial by the Southwest Oncology Group (SWOG)-0124 was conducted to confirm the results of JCOG-9511 in 651 people from North America, with similar eligibility criteria to those in the Japanese trial (8). SWOG-0124 found no significant differences between IP and EP in terms of tumor response, PFS, and OS. Thus, EP remains the standard of care for patients with ED-SCLC, at least for non-Japanese populations. In a comparison of two trials, there was no difference in the PFS of the EP group (9.4 months in JCOG-9511 vs. 9.1 months in SWOG-0124). On the contrary, for the IP group, there was a definite difference between the two studies: a median PFS of 12.8 months for JCOG-9511 and 9.9 months for SWOG-0124 (P < 0.001) (18). However, patients of male sex and with a poor performance status, who are generally regarded as having a poor prognosis, were present in larger numbers in the JCOG-9511 IP group than in the SWOG-0124 IP group. Thus, differences according to ethnicity are possible.

The most reasonable explanation for differences of irinotecan efficacy across ethnicities could be pharmacogenomic differences in the metabolism of irinotecan between Asian and Western populations. There has been no direct comparison of irinotecan metabolism-related genes and the efficacy of irinotecan in SCLC patients across geographic regions. According to Gandara et al. differences in genes involved in paclitaxel disposition or DNA repair were observed between Japanese and American patients with lung cancer (19). Also, Lampe et al. reported that the allele and genotype frequencies of *UGT1A1*, which is related to glucuronidation of a metabolite of irinotecan, varied between Asians and Caucasians (20). A specific single nucleotide polymorphism in the adenosine triphosphate-binding cassette (ABC) gene is correlated with the efficacy of irinotecan-based chemotherapy. Han et al. reported that the ABCC2-24TT and 3972TT genotypes were associated with a higher RR and longer PFS in Korean patients with advanced lung cancer (21). We infer a possible association between gene polymorphism, such as the ABC gene, and efficacy of irinotecan in SCLC. However, there have not been any reports of differences of ABC gene polymorphism according to ethnicity. Moreover, to date, UGT1A1 has not been reported as significantly correlated with irinotecan efficacy in SCLC (22, 23). Therefore, analysis of differences in genes related to the metabolism of irinotecan-based chemotherapeutics and of their direct correlation with efficacy is warranted.

In a recent phase III trial in Korean patients with ED-SCLC, although OS and PFS were not significantly different between the EP and IP arms, there was a favorable trend toward the IP regimen (OS, 10.9 months vs. 10.3 months, P = 0.120; PFS, 6.5 months vs. 5.8 months, P = 0.115). A higher RR was observed in IP (62.4% vs. 48.2%, P = 0.006) (2). Of note, the authors concluded that IP chemotherapy might be beneficial for these particular subgroups: male gender, < 65 years old, and ECOG PS 0/1 patients. In 62 Chinese patients, a randomized, prospective phase II study showed the efficacy of IP was similar to that of EP for untreated ED-SCLC; median OS was 18.1 months in IP vs.15.8 in EP (23). In a meta-analysis by Jiang et al., six randomized controlled trials involving 1,476 patients, without considering ethnicity, showed that irinotecan/platinum significantly improved the risk ratio (RR) and OS compared with etoposide/platinum with less hematological toxicity (4). In addition, in a recent meta-analysis of 12 randomized controlled trials involving 2,030 patients, including more Asian populations, the irinotecan/platinum regimen also significantly improved the 1- and 2-year survival rates of patients with previously untreated ED-SCLC (RR 1.16, 95% CI, 1.03–1.31, P = 0.02 vs RR 1.79, 95% CI, 1.22–2.61, P = 0.003, respectively) (24). Taken together, the IP regimen should be strongly considered as first-line therapy in Asian populations.

Interestingly, belotecan, a new camptothecin analog, was superior as a single agent in the first-line regimen compared to irinotecan or etoposide alone. In a preclinical study, belotecan was a more potent topoisomerase I inhibitor and had superior antitumor activity to camptothecin and topotecan (25). In a phase II study, belotecan showed a 42.9% RR and a modest OS of 11.4 months as the first-line treatment for ED-SCLC, comparable to irinotecan alone (26). Neutropenia occurred in 74% of the patients but was reversible, generally manageable, and not cumulative. There has been no confirmatory trial of the efficacy of belotecan in the first-line setting for patients medically unfit for combination chemotherapy. However, our findings will enable a confirmatory trial of the efficacy of belotecan alone compared with irinotecan, etoposide, topotecan, and paclitaxel alone.

Regarding strategies to use cytotoxic chemotherapy in the second-line setting, a consensus has not been reached on the best and most effective regimen. However, based on our results, in patients receiving combination treatment as their second-line treatment, a statistically significant increase in OS was observed compared to those receiving single agents, even in the population with the EP regimen as first-line treatment. OS did not significantly differ between single-agent regimens such as topotecan, irinotecan, belotecan, or etoposide at the second-line treatment. In a phase III study comparing topotecan alone and cyclophosphamide/adriamycin/vincristine combination therapy as second-line therapy, both groups showed similar response and survival rates, but the group receiving topotecan had less toxicity than the combination therapy group (27). Although there are limited data on similar effects between irinotecan and topotecan, this has not been evaluated in randomized studies. Meanwhile, in the Western population, there was a new selective oncogenic transcription inhibitor, lurbinectedin, which showed anti-tumor activity in 105 patients with small-cell lung cancer who had received prior platinum-based chemotherapy and had no brain metastases (28). The objective response rate of lurbinectidine was 35.2%, median progression-free survival was 3.5 months, and median overall survival 9.3 months. When it comes to our results showing no superior single agent in 2nd line setting and lubinectidine studied only in Europe and USA population, the interpretation and application of results of lurbinectedin should be with caution. There might be other metabolic and genetic differences of drugs according to ethnicity as we mentioned above.

Poor prognostic factors for patients with ED-SCLC were elderly age, male gender, COPD, normal lipids, and EP chemotherapy. COPD could be a driving factor in lung cancer, but there have been conflicting results from previous studies about whether COPD affects the survival of lung cancer patients on chemotherapy or a tyrosine kinase inhibitor (28). Recent analysis showed that COPD is an independent prognostic risk factor for lung cancer (29). The prognostic role of lipidemia in cancer patients is controversial. Hypocholesterolemia in malignancy might come from an increased demand for cholesterol from neoplastic cells, resulting in increased LDL removal (30).

Several limitations are apparent in this study and must be considered when interpreting the results. First, there may be bias in identifying LD or ED-SCLC patients using the operational definition. However, to overcome such bias we used a strict multistep approach. The operational definition showed high accuracy to differentiate LD and ED patients when validated among 357 SCLC patients at a single institution. Moreover, the survival rates of the subgroups defined were comparable to those reported previously. Second, we analyzed the HIRA data retrospectively; they do not include information on the frequency of adverse drug reactions, dose intensity, or cause of death. However, this is, to our knowledge, the largest comparative analysis in Asian patients with ED-SCLC.

## Conclusions

Authors found that IP as the first-line regimen had a significantly favorable effect on the OS and TFST of patients with ED-SCLC compared to EP. Among the single agents, belotecan showed a superior OS to irinotecan or etoposide alone. As the second-line therapy, combination chemotherapy had clinical benefits over single agents; there were no significant differences among the single agents.

## Data Availability Statement

The datasets generated during and/or analysed during the current study are not publicly available due to Data Protection Laws and Regulations in Korea, but final analyzing results are available from the corresponding authors on reasonable request.

## Ethics Statement

The studies involving human participants were reviewed and approved by the Uijeongbu St. Mary Hospital of the Catholic University of Korea and HIRA. Written informed consent for participation was not required for this study in accordance with the national legislation and the institutional requirements.

## Author Contributions

YKo, JL, and HL conceptualized the work. JL, YKi, JH, YKo, HL, SK, and S-YS acquired, analyzed, and interpreted the data. YKo, JL, and JH have drafted the work. JL, JH, and YKo substantively revised it. JH and YKo were co-corresponding authors in this work. All authors contributed to the article and approved the submitted version.

## Conflict of Interest

The authors declare that the research was conducted in the absence of any commercial or financial relationships that could be construed as a potential conflict of interest.

## Supplementary Material

Supplementary Table 1 Sensitivity, specificity, and accuracy for prediction of LD- or ED-SCLC using the operational definition. LD, limited-stage disease; ED, extensive-stage disease; SCLC, small cell lung cancer; PPV, positive predictive value; NPV, negative predictive value.

Supplementary Figure 1 Kaplan–Meier curve for overall survival (OS) between extensive and limited stage disease small cell lung cancer (ED-SCLC and LD-SCLC, respectively) patients who underwent systemic treatment.

Supplementary Figure 2 Kaplan–Meier curve for time to first subsequent therapy (TFST) **(A)** and overall survival (OS) **(B)** of the combination chemotherapy (combination-chemotherapy group [CG]) and the single-agent group [SG]); **(C)** OS of patients who received belotecan, etoposide, and irinotecan as a single agent as first-line treatment of ED-SCLC.

Supplementary Figure 3 Kaplan–Meier curve for overall survival (OS) of the combination chemotherapy (combination-chemotherapy group [CG]) and the single-agent group [SG]) as the second-line regimen in the total population **(A)** and post-EP chemotherapy population **(B)**.
