# Supplementary material for: Treatment Outcomes of 9,994 Patients With Extensive-Disease Small-Cell Lung Cancer From a Retrospective Nationwide Population-Based Cohort in the Korean HIRA Database
Source: Front Oncol. 2021 Mar 22;11:546672. doi: 10.3389/fonc.2021.546672 (PMC8019929; doi:10.3389/fonc.2021.546672)
Supplement: Supplementary Table 1 — Sensitivity, specificity, and accuracy for prediction of LD- or ED-SCLC using the operational definition. LD, limited-stage disease; ED, extensive-stage disease; SCLC, small cell lung cancer; PPV, positive predictive value; NPV, negative predictive value. [file Table_1.docx]

**Supplementary Table 1**. Sensitivity, specificity, and accuracy for prediction of LD- or ED-SCLC using the operational definition.

|  | Sensitivity (%) | Specificity (%) | PPV (%) | NPV (%) | Accuracy (%) |
| --- | --- | --- | --- | --- | --- |
| Operational definition | 100 | 64.6 | 85.4 | 100 | 88.52 |

Abbreviations: LD, limited-stage disease; ED, extensive-stage disease; SCLC, small cell lung cancer; PPV, positive predictive value; NPV, negative predictive value.
