# Supplementary figures and images for: Treatment Outcomes of 9,994 Patients With Extensive-Disease Small-Cell Lung Cancer From a Retrospective Nationwide Population-Based Cohort in the Korean HIRA Database
Source: Front Oncol. 2021 Mar 22;11:546672. doi: 10.3389/fonc.2021.546672 (PMC8019929; doi:10.3389/fonc.2021.546672)

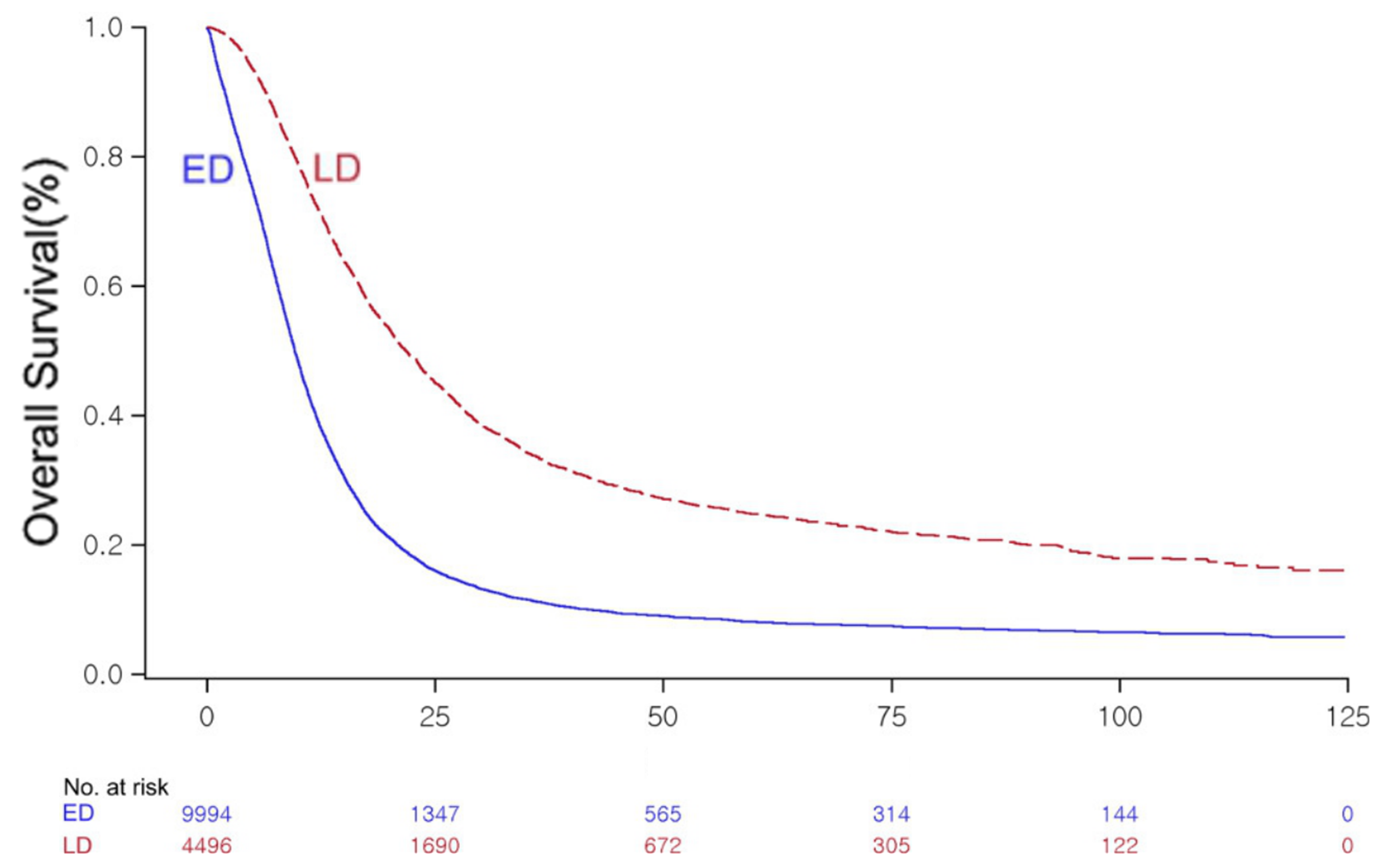

Supplement: Supplementary Figure 1 — Kaplan–Meier curve for overall survival (OS) between extensive and limited stage disease small cell lung cancer (ED-SCLC and LD-SCLC, respectively) patients who underwent systemic treatment. [file Image_1.tif]

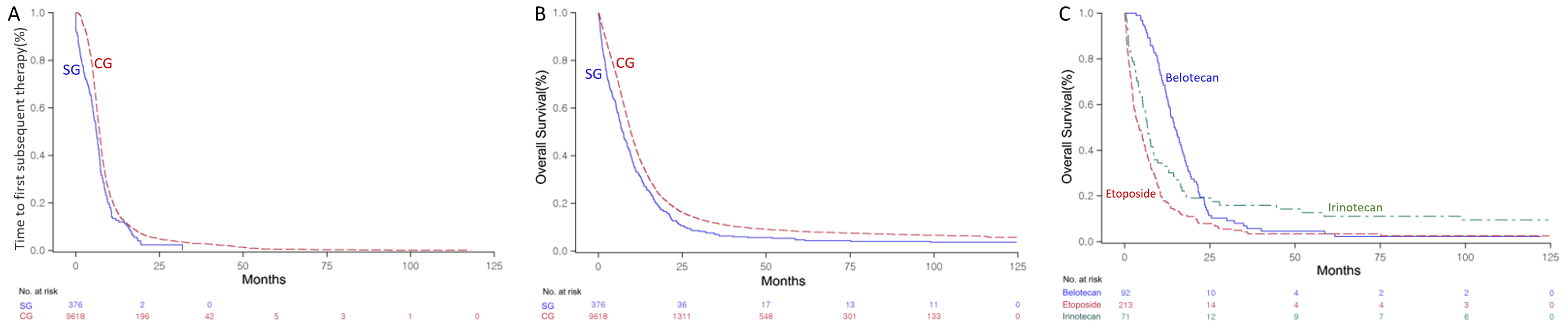

Supplement: Supplementary Figure 2 — Kaplan–Meier curve for time to first subsequent therapy (TFST) (A) and overall survival (OS) (B) of the combination chemotherapy (combination-chemotherapy group [CG]) and the single-agent group [SG]); (C) OS of patients who received belotecan, etoposide, and irinotecan as a single agent as first-line treatment of ED-SCLC. [file Image_2.tif]

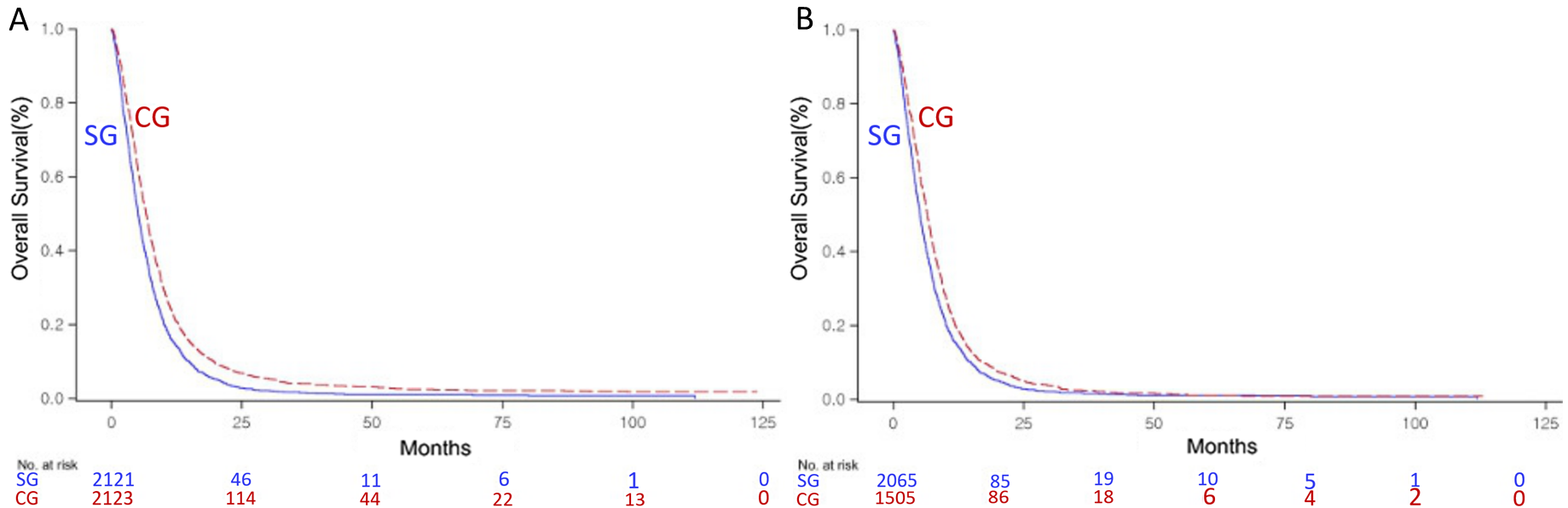

Supplement: Supplementary Figure 3 — Kaplan–Meier curve for overall survival (OS) of the combination chemotherapy (combination-chemotherapy group [CG]) and the single-agent group [SG]) as the second-line regimen in the total population (A) and post-EP chemotherapy population (B). [file Image_3.tif]
